# Executive functioning in the first 24 months: A scoping review of the A-not-B task

**DOI:** 10.1007/s00426-026-02330-5

**Published:** 2026-07-15

**Authors:** Cláudia Ramos, Alfredo F. Pereira, Joana Baptista

**Affiliations:** 1 Centro de Investigação e Intervenção Social, Iscte-Instituto Universitário de Lisboa, Lisboa, Portugal; 2 Center for Psychology at the University of Porto, Faculty of Psychology and Education Sciences, University of Porto, Porto, Portugal

## Abstract

Recent studies highlight the importance of early executive functioning (EF); notwithstanding, these studies remain limited due to methodological difficulties. The A-not-B task has been adapted to assess EF; however, variations in its administration hinder result comparability and standardization. This scoping review mapped the procedures used in studies employing the A-not-B task to assess EF in children up to 24 months. Searches were conducted in PubMed, Scopus, and Web of Science for empirical studies published in English. From 350 references, 53 articles were included. Most studies primarily focused on assessing global EF, working memory, and inhibitory control; employed the standard two-locations task with fixed delays, used two consecutive correct trials to switch locations, and measured accuracy as the main outcome. Testing typically ended after the completion of trials/sequences. Few studies reported inclusion criteria. This review summarizes A-not-B procedures to identify research gaps and support methodological consistency in early EF assessment.

**Supplementary Information:**

The online version contains supplementary material available at https://doi.org/10.1007/s00426-026-02330-5.

Executive functioning (EF) refers to a set of higher-order cognitive abilities that support flexible, goal-directed behavior and underlie early learning, self-regulation, and socio-emotional development (Diamond, 2013). Among its core components, working memory, involves the capacity to maintain and manipulate information over short periods; cognitive flexibility enables shifting between rules or perspectives in response to changing environmental demands; and inhibitory control, encompasses the ability to suppress prepotent responses, in favour of more adaptive ones (e.g., Diamond, 2013; Miyake et al., 2000).

A compelling body of research demonstrates that individual differences in EF during early childhood are consistently associated with a wide range of developmental outcomes, including academic achievement (Zelazo & Carlson, 2020), social competence (Kochanska et al., 2000), emotion regulation (Carlson & Wang, 2007), and overall health and well-being (Moffitt et al., 2011). Conversely, early EF difficulties predict greater peer problems throughout childhood (Holmes et al., 2016), and are linked to conditions such as attention-deficit hyperactivity disorder (ADHD) and autism spectrum disorder (ASD) (Ozonoff et al., 1991; Rommelse et al., 2011).

These associations highlight the need to examine EF as early as possible in development. Evidence shows that emerging EF difficulties can already be observed by 9 months of age in high-risk populations (e.g., Lipina et al., 2005; Sun et al., 2009). Early detection is particularly important because interventions introduced during periods of heightened neural plasticity may alter developmental trajectories more effectively (Wass, 2015). This is supported by evidence from children exposed to psychological deprivation, who show developmental gains when placed early in enriched caregiving environments such as foster or adoptive care (Colvert et al., 2008; Moulson et al., 2009). Since EF develops gradually yet individual differences remain relatively stable (Best & Miller, 2010; Miyake & Friedman, 2012), assessing EF from infancy represents a crucial step for identifying developmental difficulties before they consolidate. Reliable assessments in infancy are therefore essential, both for improving early clinical identification and for advancing scientific understanding of EF’s developmental course.

## Early development of executive functioning

The emergence of core EF components within the first years of life strengthens the rationale for early assessment.

Inhibitory control abilities appear to emerge around the age of 5 to 6 months (Cuevas et al., 2012a; Holmboe et al., 2018; MacNeill et al., 2018), infants withhold gaze towards distractors (Holmboe et al., 2018) and pause actions in response to caregivers’ instructions (Kochanska et al., 1998). Until the end of the first year of life, infants exhibit steady improvements in their inhibitory abilities (Diamond, 2013).

Similarly, regarding their working memory abilities, infants display rudimentary abilities as young as five, six months (e.g., Cuevas & Bell, 2011; Cuevas et al., 2012a), showing a steady increase in their abilities from 7.5 to 12 months of age (Diamond, 2013). Twelve-month-olds tolerate delays of up to 10 s (Diamond, 1985, 1990), and by 18 months, children retrieve objects from one of four possible locations after a delay and with around 75% accuracy (Garon et al., 2014).

Higher-level cognitive abilities developed later; e.g., 2-year-old children are able to regulate their behavior by following and integrating simple rules (Best & Miller, 2010; Cuevas et al., 2017).

Neuroscientific evidence supports this developmental trajectory. Myelination of frontal axons begins around 6–8 months of age (Deoni et al., 2011), and coincides with emerging inhibition and working memory (e.g., Cuevas et al., 2012a). Structural and functional changes in the dorsolateral prefrontal cortex—such as dendritic growth between 7 and 12 months of age, and increased frontal EEG activity—correspond with improvements in the A-not-B performance (Bell & Fox, 1992, 1997). Further studies show that inhibitory control in 9–10-month-olds is linked to frontal cortical activity (Cuevas et al., 2012b; Holmboe et al., 2018), with 10-month-olds displaying prefrontal and parietal activation during tasks tapping inhibition (Fiske et al., 2022). The first year of life is therefore a period of pronounced neural reorganization, including synaptic pruning, myelination (Huttenlocher, 2002), and a rapid increase in brain volume—from 36% of adult size at 2–4 weeks to 72% by 12 months (Knickmeyer et al., 2008). The onset of crawling at about 5 months is associated with EEG coherence changes (Bell & Fox, 1996), and frontal myelination accelerates around 6 months (O’Muircheartaigh et al., 2014), further underscoring the rapid neural maturation that supports emerging EF skills.

### The A-not-B task: a window into early executive functioning

Despite the importance of early EF, studies in infancy remain comparatively limited, partly due to challenges in assessing preverbal children and capturing emerging cognitive abilities (e.g., Cuevas et al., 2012a, 2012b; Holmboe et al., 2008; Holmboe et al., 2018). Few tasks have been designed for this developmental window (Hendry et al., 2022), though recent efforts include the Freeze-Frame task (Holmboe et al., 2008) and the Early Childhood Inhibitory Touchscreen Task (Hendry et al., 2022), both targeting inhibitory control. Existing measures typically isolate a single EF component; yet the multidimensional nature of EF calls for measures that more holistically engage multiple processes (Banich, 2009).

Among the tools developed for infants, the A-not-B task has become one of the most widely used indicators of early EF (Cuevas et al., 2017). Originally developed by Piaget (1954) to study object permanence, the task involves repeatedly hiding a toy at location A (non-reversal trials) and then switching to location B (reversal trials). Infants between 8 and 12 months often commit the so-called A-not-B error—reaching back to location A despite seeing the toy hidden at B. Successful performance requires maintaining and updating the object’s location (working memory), as well as inhibiting a previously reinforced motor response (inhibitory control). Shifting from A to B may also draw on early forms of cognitive flexibility, positioning the A-not-B task as a multidimensional index of emerging EF skills during a period when infants increasingly organize attention and coordinate actions with caregivers (e.g., Gago Galvagno et al., 2019).

Performance on the A-not-B task is shaped by several developmental and procedural factors: older infants typically show fewer perseverative errors; longer delays between hiding and retrieval increase the likelihood of errors; and increasing the number of hiding locations often reduces perseveration (Lipina et al., 2005; Marcovitch & Zelazo, 1999; Wellman et al., 1986). Task format also matters, and there is a reaching and a looking version of the A-not-B. The looking and reaching versions of the A-not-B task differ primarily in the observable behaviour that is used to decide if there was perseveration. In the reaching version, it is the movement of reaching towards one location, while in the looking version, it is the fixation. Looking versions rely on oculomotor control, whereas reaching versions additionally recruit motor planning, though both engage attention, working memory, and inhibitory control skills (Bell & Adams, 1999). Findings comparing these formats are mixed: some studies report better performance in looking versions (Hofstadter & Reznick, 1996; Diamond, 1988), others find comparable outcomes (Matthews et al., 1996), and still others highlight divergences across error types (Bell & Adams, 1999). The results underscore the sensitivity of the A-not-B task to methodological variation and suggest that procedural features may modulate the cognitive demands placed on infants.

Theoretical accounts of the A-not-B error have also evolved. Although Piaget attributed the A-not-B error to a transitional understanding of object permanence, subsequent accounts have emphasized attention (Horobin & Acredolo, 1986), memory–attention interactions (Perner, 1991), or distinctions between long- and short-term memory encoding (Harris, 1989; Sophian & Wellman, 1983). Dynamic systems models further emphasize that performance reflects an interaction of cognitive, motor, and visuospatial processes (Smith et al., 1999; Spencer et al., 2001). Contemporary perspectives, however, explicitly link A-not-B performance to EF (Diamond, 2013), with reversal errors reflecting inhibitory control demands (Bell & Adams, 1999) and non-perseverative errors reflecting attentional limitations (Diamond & Goldman-Rakic, 1989). Neurodevelopmental evidence corroborates this view, linking A-not-B performance to maturation of prefrontal systems critical for EF (Diamond & Goldman-Rakic, 1989; Hodel, 2018).

### Aim and structure of the scoping review

Despite its theoretical significance and widespread use, the A-not-B task shows considerable variability in design and methodology across studies. Even subtle procedural differences can markedly influence the cognitive and regulatory demands placed on infants, influencing the probability of observing the A-not-B error, thus shaping how results are interpreted across studies. In light of this challenge, the present scoping review aims to map the procedures used to administer the A-not-B task in studies of children up to 24 months of age, as well as related constructs—such as executive attention and executive control—often examined alongside EF in infancy. Executive attention is conceptualized as a top-down regulatory mechanism of attentional control (Posner & DiGirolamo, 1998) closely linked to EF, while executive control describes the top-down coordination of cognitive and behavioural processes in a goal-directed manner (Nigg, 2017). There is a notable overlap with core EF components, since these abilities are assessed using the same measures (e.g., the A-not-B task).

To achieve this aim, the review systematically identifies and synthesizes the methodological approaches used across studies, providing an overview of how the A-not-B task has been implemented to assess early EF. By bringing together these disparate procedures, the review highlights areas of consistency and divergence that currently limit comparability across studies. Given the rapid neurocognitive changes that characterize the first two years of life, clarifying the methodological foundations of the A-not-B task is particularly important. Ultimately, this synthesis may support movement toward greater consensus in the administration and interpretation of the A-not-B task as a measure of early EF, thereby helping guide future studies in the field.

## Method

The present scoping review followed the PRISMA-ScR (Preferred Reporting Items for Systematic Reviews and Meta-Analyses extension for Scoping reviews) guidelines (Tricco et al., 2018). This study was not preregistered.

### Search strategy

We conducted a comprehensive search for all studies published up to December 31, 2024, across the following databases: PubMed, Scopus, and Web of Science. The keywords “A-not-B AND (infant* OR child*)” were applied across each database to identify relevant literature. The search was limited to empirical studies published in English. Further studies were identified by manually searching the reference lists of pertinent articles.

### Inclusion and exclusion criteria

The inclusion criteria considered the following: (a) empirical studies; (b) studies that assessed infants and children up to 24 months of age; and (c) studies that administered the A-not-B task to assess EF or related concepts, such as attention, executive attention, or executive control. The exclusion criteria considered the following: (a) meta-analyses, systematic and non-systematic reviews, randomized controlled trials, and case studies; and (b) studies that assessed other cognitive abilities other than EF or related concepts, such as object permanence and cognitive development.

### Study selection

The records that were retrieved through the systematic search of the databases were exported to Rayyan, a software designed to facilitate literature reviews (Rayyan Systems Inc., 2023). The removal of duplicate literature was conducted using a standard function in Rayyan; afterwards, titles and abstracts were reviewed. Two independent raters reviewed these to exclude articles that did not meet the inclusion criteria for the scoping review. Disagreements were resolved by consensus. Inter-rater reliability was assessed using Cohen’s Kappa, indicating excellent agreement (κ = 0.852, *p* <.001). Articles meeting the inclusion criteria proceeded to the next stage—i.e., full-text screening.

### Data charting and synthesis

A data-extraction form was developed, incorporating variables deemed pertinent to the research questions and study characteristics. The included variables were: authors; year of publication; study population; abilities assessed; inclusion criteria; the A-not-B version used to measure EF or related concepts; the format of the task (looking and/or reaching); the number of locations; delay between hiding and searching; criteria for switching between locations; dependent measures; and the criteria for the end of testing.

The results are presented below, organized according to the following: (a) researchers’ assessment interests when administering the A-not-B task; (b) the most common procedures administered; (c) specific administration procedures; (d) researchers’ scoring methods of the children’s performance; and (e) the participant inclusion criteria used for the analysis.

## Results

**Fig. 1:**
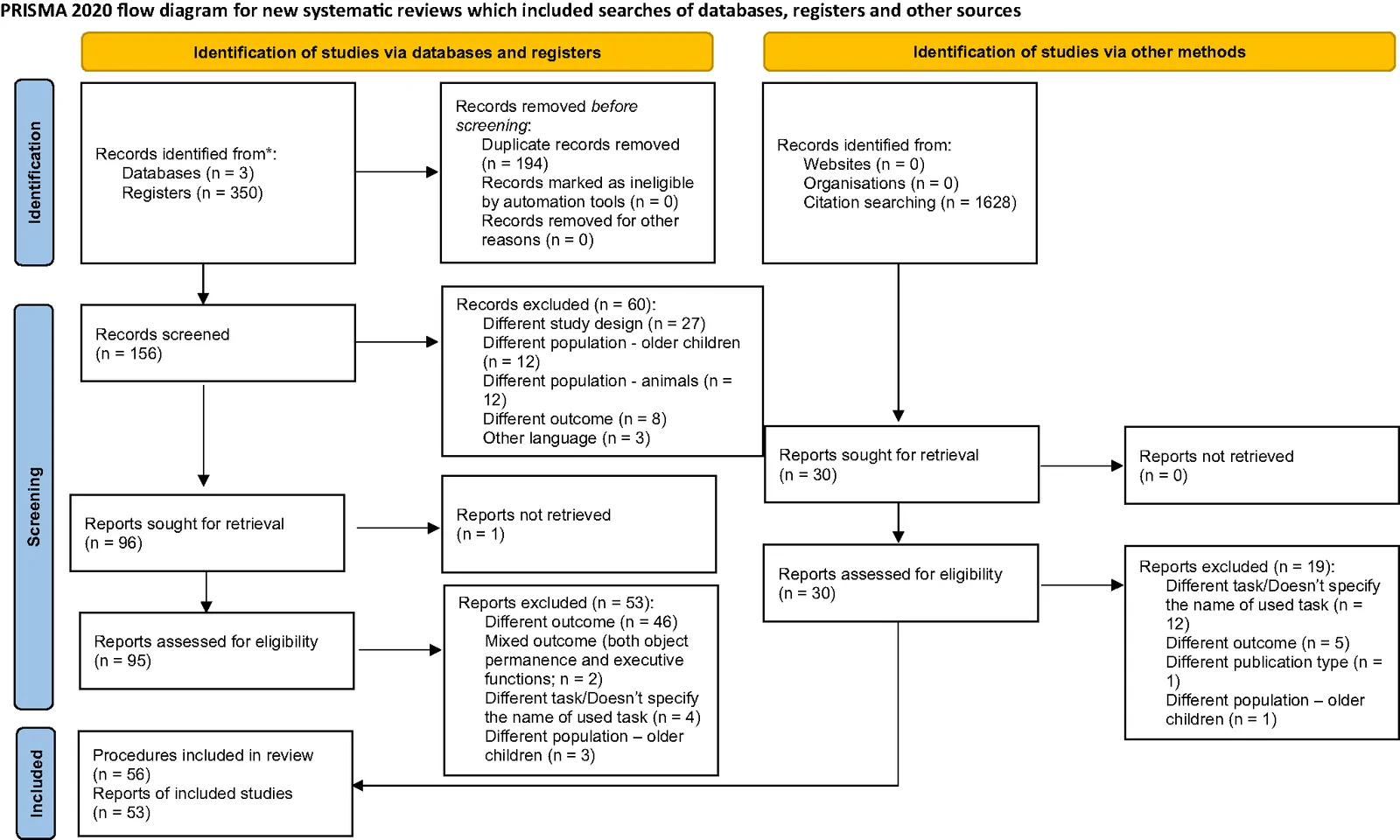
PRISMA flow diagram of study selection procedure

The initial search of electronic databases yielded 350 records (Fig. 1). After removing 194 duplicates, 60 records were excluded based on title and abstract screening. On the full-text screening, one article was not retrieved, and a further number of 53 records were excluded by not meeting the inclusion criteria. An additional 11 studies were included through reference checking, resulting in a total of 53 eligible papers, corresponding to 56 procedures, given that three articles described two different A-not-B procedures each (See Tables 1 and 2 for a synthesis of the included studies and details of the corresponding administration procedures). The results presented below correspond to a total of 56 reported procedures reported in the final set of papers selected.

**Table 1:** Results from the scoping review per study: Study characteristics and task information

Author, year	Study population	Abilities assessed	Version of the task	Task format	# Locations	Training	Delay	Switch between locations
Abubakar et al., 2013	6 to 35 months	EF	Standard A-not-B task	Reaching	2	No	Fixed delay	2 consecutive correct trials
Bacher et al., 2017	10 months	WM	Standard A-not-B task	Looking	2	No	No delay	2 consecutive correct trials
Bell & Wolfe, 2007	8 months	WM and IC	Standard A-not-B task	Looking	2	No	No delay	2 consecutive correct trials
Bell, 2012	8 months	WM	Standard A-not-B task	Looking	2	No	No delay	2 consecutive correct trials
Blankenship et al., 2019	10 months	EF	Standard A-not-B task	Looking	2	No	No delay	2 consecutive correct trials
Borioni et al. 2022	13 to 22 and 15 to 24 months	CF	Clearfield et al., 2006	Reaching	2	No	Fixed delay	Established sequence
Carlson et al., 2004	24 months	EF	ABMHL	Reaching	5	Yes	Fixed delay on reversal trials	3 consecutive correct trials
Clearfield & Niman, 2012	6, 9 and 12 months	CF	Clearfield et al., 2006	Reaching	2	No	Fixed delay	Established sequence
Colombo et al., 2014	9 and 12 months	WM and IC	Standard A-not-B task	Reaching	2	No	Increasing fixed delay	Not established
Cuevas & Bell, 2011	5 to 10 months	WM	Standard A-not-B task	Looking	2	No	Varies according to performance: if criterion is achieved, children were then tested with a delay.	2 consecutive correct trials
Cuevas & Bell, 2014	24 months	EF	ABID	Looking	2	No	Fixed delay	2 consecutive correct trials
Cuevas et al., 2012a	5 to 10 months	WM	Standard A-not-B task	Looking	2	No	Varies according to performance: if criterion is achieved, children were then tested with a delay.	2 consecutive correct trials
Cuevas et al., 2012b	10 months	WM and IC	Standard A-not-B task	Looking	2	No	Varies according to performance: if criterion is achieved, children were then tested with a delay.	2 consecutive correct trials
Espy et al., 1999a	17 to 21 months	EF	Standard A-not-B task	Reaching	2	No	Fixed delay	2 consecutive correct trials
Espy et al., 1999b	23 to 66 months	EF	Standard A-not-B task	Reaching	2	No	Fixed delay	2 consecutive correct trials
Frick et al., 2018	18 months	EF	Standard A-not-B task	Reaching and looking	2	No	Fixed delay	Established sequence
Gago Galvagno et al., 2019	18 to 24 months	EF	ABMHL	Reaching	5	No	Fixed delay	A trials were repeated until the child found the object on A 3 times.
Hendry et al., 2022	10 and 16 months	IC	Standard A-not-B task	Reaching	2	No	No delay	2 or 3 consecutive correct trials (depending on age)
Hogan et al., 2013	9 and 12 months	EF	Standard A-not-B task	Reaching	2	No	Fixed delay	2 consecutive correct trials
Holmboe et al., 2008	9 months	IC	Standard A-not-B task	Reaching	2	No	Varies according to performance	2 consecutive correct trials
Holmboe et al., 2018	9 months	IC	Standard A-not-B task	Reaching	2	No	Varies according to performance	2 consecutive correct trials
Ishibashi & Moriguchi, 2017	16 to 37 months	IC	Standard A-not-B task	Reaching	2	No	No delay	Not established
Jia et al., 2021	8 months	WM, distractibility, and IC	Standard A-not-B task	Reaching	1 to 3	No	Varies according to performance	Not established
Jia et al., 2022	8 months	WM and attention	Standard A-not-B task	Reaching	1 to 3	No	Varies according to performance	Not established
Johansson et al., 2014	10 months	CF	Clearfield et al., 2006	Reaching and looking	2	No	Fixed delay	Established sequence
Johansson et al., 2015	12 to 13 months and 24 to 25 months	EF	Clearfield et al., 2006	Looking	2	No	Fixed delay	Established sequence
Karonen et al., 2023	8 months	EF	Standard A-not-B task	Reaching	2	No	Varies according to performance: Delay levels	Established sequence
Lipina et al., 2005	6 to 14 months	EF	Standard A-not-B task	Reaching	2	No	Varies according to performance	2 consecutive correct trials
Miller & Marcovitch, 2015	14 to 18 months	EF	ABMHL	Reaching	5	Yes	Fixed delay	3 consecutive correct trials
Mulder et al., 2020	14 months	EF	ABMHL	Reaching	4	Yes	Fixed delay	2 consecutive correct trials
Nampijja et al., 2012	15 months	EF	Standard A-not-B task	Reaching	2	No	Fixed delay	2 consecutive correct trials
Nimmapirat et al., 2024	12 and 18 months	Cool EF	Standard A-not-B task	Looking	2	No	Varies according to performance: if criterion is achieved, children were then tested with a delay.	2 consecutive correct trials
Noland et al., 2003	9.5 to 12.5 months	EF	Standard A-not-B task	Reaching	3	Yes	Varies according to performance	2 consecutive correct trials
Nolvi et al., 2018	8 months	EF	Standard A-not-B task	Reaching	2	No	Varies according to performance: Delay levels	Established sequence
Phillips et al., 2011	18 to 22 months	WM and IC	Standard A-not-B task	Reaching	1 to 2	No	Fixed delay	Not established
Prado et al., 2016a	18 months	EF	Standard A-not-B task	Reaching	2	No	Fixed delay	2 consecutive correct trials
Prado et al., 2016b	18 months	EF	Standard A-not-B task	Reaching	2	No	Fixed delay	2 consecutive correct trials
Prado et al., 2016c	18 months	EF	Standard A-not-B task	Reaching	2	No	Fixed delay	2 consecutive correct trials
Prado et al., 2016d	18 months	EF	Standard A-not-B task	Reaching	2	No	Fixed delay	2 consecutive correct trials
Prado et al., 2018	18 months	EF	Standard A-not-B task	Reaching	2	No	Fixed delay	2 consecutive correct trials
Pushina et al., 2005	7 to 12 months	WM	Standard A-not-B task	Reaching	2	Yes	Varies according to performance: if criterion is achieved, children were then tested with a delay.	Established sequence
Pyykko et al., 2020	18 months	WM	Standard A-not-B task	Reaching	2	No	Fixed delay	2 consecutive correct trials
Shinya & Ishibashi, 2022	12 months	Perseverative attention and sustained attention	Clearfield et al., 2006	Looking	2	No	Fixed delay	Established sequence
St. John et al., 2016	12 and 24 months	WM and IC	Standard A-not-B task	Reaching	2	No	Varies according to performance: Delay levels	2 consecutive correct trials
Sun et al., 2009	8 months	WM and distractibility	Standard A-not-B task	Reaching	1 to 3	No	Varies according to performance: Delay levels	2 consecutive correct trials
Tofail et al., 2018	24 months	WM	Standard A-not-B task	Reaching	2	No	Fixed delay	Not established
Toth et al., 2008	17–24 months	WM and IC	Standard A-not-B task	Reaching	2	Yes	Varies according to performance: Delay levels	2 consecutive correct trials
			ABID	Reaching	2	Yes	Fixed delay	2 consecutive correct trials
Tsetlin et al., 2012	10 to 11 months	WM	Standard A-not-B task	Reaching	2	No	Varies according to performance	2 consecutive correct trials
van de Weijer-Bergsma et al., 2010	7 and 10 months	EF	Standard A-not-B task	Reaching and looking	2	Yes	Varies according to performance	2 consecutive correct trials
	14 months	EF	ABID	Reaching	2	No	Varies according to performance	2 consecutive correct trials
van de Weijer-Bergsma et al., 2016	7 and 10 months	EF	Standard A-not-B task	Reaching and looking	2	Yes	Varies according to performance	2 consecutive correct trials
	14 months	EF	ABID	Reaching	2	No	Varies according to performance	2 consecutive correct trials
Wang et al., 2021	5 to 7 months	IC	Clearfield et al., 2006	Reaching	2	No	No delay	Established sequence
Wiebe et al., 2010	15 and 20 months	Executive control	ABID	Reaching	2	Yes	Fixed delay	2 consecutive correct trials
Zmyj et al., 2015	18 months	WM	Standard A-not-B task	Reaching	2	No	Fixed delay	4 successful retrievals from A

**Table 2:** Results from the scoping review per study: End of Testing, scoring, data on children’s performance, role of age, and inclusion criteria

Author, year	End of testing	Scoring	Data on Performance	Role of children’s age	Inclusion criteria
Abubakar et al., 2013	End of trials	Total correct, perseverative errors, maximum error run	cf. Abubakar et al., 2013	Age was significantly related to total correct (*r* =.467, *p* <.001) and maximum error run (*r* = − **.3**69, *p* <.001).	All ten trials
Bacher et al., 2017	2 consecutive errors on reversal trials	Total correct	High performers: *M*(*SD*) = 0.80(0.06) Low performers: *M*(*SD*) = 0.40(0.11)	Not applicable	Performance that includes correct and incorrect trials and trials that lasted at least 3 min
Bell & Wolfe, 2007	Incorrect responses in two reversal trials	Total correct	-	Not applicable	Not established
Bell, 2012	Incorrect responses in two reversal trials	Perseverative and non-perseverative errors	High WM group: 59% correct performance on non-reversal trials (range = 24%–91%) and 64% on reversal trials (range = 33%–100%). Low WM group: 38% correct performance on non-reversal trials (range = 0%–67%) and 7% on reversals (range = 0%–33%).	Not applicable	Not established
Blankenship et al., 2019	Not established	Perseverative and non-perseverative errors (proportion correct)	*M*(*SD*) = 0.58(0.017)	Not applicable	Not established
Borioni et al. 2022	End of trials	Total correct, perseverative errors, speed	Total correct (% errors): Baseline - *M*(*SD*) = 30(15.7); Post-intervention - *M*(*SD*) = 22.2(23.3) Perseverative errors (n): Baseline - *M*(*SD*) = 0.6(0.5); Post-intervention - M(SD) = 0.03(0.05) Speed: Baseline (seconds) - *M*(*SD*) = 4.0(2.8); Post-intervention - M(SD) = 3.6(3.1)	Not applicable	Not established
Carlson et al., 2004	Correct response on reversal trials	Perseverative errors	*M*(*SD*) = 3.26(1.06)	Not applicable	Completion of more than half of test trials
Clearfield & Niman, 2012	End of trials	Percentage of infants reaching A on B1 and Profile	cf. Clearfield and Niman (2012)	Higher-SES infants perseverated significantly more at 6 and 9 months (6 months: *X*^2^(1) = 3.35, *p* =.05; 9 months: *X*^2^(1) = 6.10, *p* <.05), whereas at 12 months, lower-SES infants showed greater perseveration, *X*^2^(1) = 3.95, *p* <.05.	Not established
Colombo et al., 2014	Not established	Maximum delay	9-month-old: *M*(*SD*) = 3.17(0.30) 12-month-old: *M*(*SD*) = 4.4(0.30)	Significant effect of age (*p* <.001)	Not established
Cuevas & Bell, 2011	Incorrect responses in two out of three reversal trials	Total correct	-	Age-related increases in EEG power and coherence, and decreases in heart rate.	Not established
Cuevas & Bell, 2014	Not established	Total correct (proportion correct)	*M*(*SD*) = 0.65(0.22)	Not applicable	Not established
Cuevas et al., 2012a	Incorrect responses in two out of three reversal trials	Total correct, perseverative and non-perseverative errors (proportion)	Total correct: 5 months - *M*(*SD*) = 0.54(0.18); 10 months - *M*(*SD*) = 0.64(0.17) Perseverative errors: 5 months - *M*(*SD*) = 0.27(0.39); 10 months - *M*(*SD*) = 0.33(0.36) Non-perseverative errors: 5 months - *M*(*SD*) = 0.58(0.22); 10 months - *M*(*SD*) = 0.69(0.19)	Main effect of age for Total correct and Non-perseverative errors, *F*(1, 110) = 14.72, *p* <.001, and *F*(1, 110) = 14.51, *p* <.001 and, respectively.	Not established
Cuevas et al., 2012b	Incorrect responses in two out of three reversal trials	Perseverative and non-perseverative errors (proportion)	Perseverative errors: *M*(*SD*) = 0.41(0.35) Non-perseverative errors: *M*(*SD*) = 0.67(0.19)	Not applicable	Not established
Espy et al., 1999a	End of trials	Perseverative errors, perseverative runs, and the number of times the child had two consecutive correct responses	Perseverative errors: *M*(*SD*) = 2.35(1.62), range = 0–4; Perseverative runs: *M*(*SD*) = 1.59(1.28), range = 0–4; No. times of two consecutive correct responses: *M*(*SD*) = 3.47(1.28), range = 1–5	Not examined	All ten trials
Espy et al., 1999b	End of trials	Total correct, number of correct consecutive responses, perseverative errors, and perseverative runs	Total correct: *M*(*SD*) = 8.20(1.60), range = 3–10; No. correct consecutive responses: *M*(*SD*) = 5.78(2.75), range = 2–10; Perseverative errors: *M*(*SD*) = 1.51(1.34), range = 0–7; Perseverative runs: *M*(*SD*) = 1.07(0.94), range = 0–5.	Main effect of age for total correct (*F*(1,114) = 22.17, *p* <.001), number of correct consecutive responses (*F*(1,114) = 29.68, *p* <.001), perseverative errors (*F*(1,114) = 35.25, *p* <.001), and perseverative runs (*F*(1,114) = 18.64, *p* <.001).	All ten trials
Frick et al., 2018	End of trials	Mean number of correct searches on all trials	*M*(*SD*) = 5.81(2.10)	Not applicable	Have codable behaviors on at least one trial
Gago Galvagno et al., 2019	After finding object in B two times	Perseverative errors, and searching correctly in B twice	*M*(*SD*) = 3.60(3.96)	For correlations, a composite of different EF tasks was used. Age was positive and significantly correlated with EF performance (*r* =.28, *p* =.046).	Not established
Hendry et al., 2022	End of trials	Correct switching, accuracy	Switching: 10 M - *M*(*SD*) = 0.187(0.164); 16 M - *M*(*SD*) = 0.179(0.115) Accuracy: 10 M - *M*(*SD*) = 0.720(0.426); 16 M - *M*(*SD*) = 0.884(0.380)	Not examined due to protocol differences between time-points	A minimum of 10 trials, one correct reach to each location
Hogan et al., 2013	Not established	Profile^a^	*Control group* 9 M: Partial − 2 infants; Complete − 2 infants; A-not-B: 6 infants; AB − 2 infants 12 M: Partial − 0 infants; Complete − 2 infants; A-not-B: 5 infants; AB − 3 infants *Sickle cell anemia* 9 M: Partial − 5 infants; Complete − 2 infants; A-not-B: 3 infants; AB − 4 infants 12 M: Partial − 0 infants; Complete − 1 infant; A-not-B: 5 infants; AB − 5 infants	No differences for the control group. Significant differences for children with sickle cell anemia between 9 and 12 months (*p* =.024)	Not established
Holmboe et al., 2008	End of trials	Maximum observed delay and cumulative measure in change trials	Maximum observed delay: *M*(*SD*) = 4.00(2.56); range = 0.00–8.61 Cumulative measure: *M*(*SD*) = 0.43(0.40); range = 0.99–1.26.	Not applicable	Not established
Holmboe et al., 2018	End of trials	Maximum delay in change trials	*M*(*SD*) = 1.35(1.44)	Not applicable	At least 10 trials and change trials
Ishibashi & Moriguchi, 2017	End of trials	Total correct	16-20M: *M*(*SD*) = 4.25(1.22); 21-25M: *M*(*SD*) = 4.57(1.90); 26-31M: *M*(*SD*) = 4.62(1.51); 32-37M: *M*(*SD*) = 5.00(1.97).	No significant main effect of age (F(3,25) = 0.22, *p* >.10)	All six trials
Jia et al., 2021	Not established	Total correct, number of times the child continued to play with the lid more than 2 s in correct trials, correct switching	-	Not applicable	Not established
Jia et al., 2022	Not established	Total correct, number of times the child continued to play with the lid more than 2 s in correct trials	-	Not applicable	Not established
Johansson et al., 2014	End of trials	Profile	cf. Johansson et al. (2014)	Not applicable	Infants not strong side bias
Johansson et al., 2015	End of trials	Looking time	*12-13M*: Correct looking no distractor: *M*(*SD*) = 0.52(0.33); Incorrect looking no distractor: *M*(*SD*) = 0.19(0.23); Correct looking with distractor: *M*(*SD*) = 0.35(0.30); Incorrect looking with distractor: *M*(*SD*) = 0.19(0.22). *24-25M*: Correct looking no distractor: *M*(*SD*) = 0.66(0.33); Incorrect looking no distractor: *M*(*SD*) = 0.10(0.20); Correct looking with distractor: *M*(*SD*) = 0.46(0.30); Incorrect looking with distractor: *M*(*SD*) = 0.07(0.13).	Main effect of age, *F*(1, 45) = 7.99, *p* =.007.	Completing at least two A trials, and one B trial, and having looking times to the correct location in A trials than to the incorrect location.
Karonen et al., 2023	Incorrect responses in more than a half of the trials	Total correct	*M*(*SD*) = 4.82(3.58); possible range = 0–18	Not applicable	Not established
Lipina et al., 2005	Five consecutive incorrect trials	Total correct, consecutive correct responses, perseverative errors, non-perseverative errors, number of A trials needed to achieve two consecutive correct responses	cf. Lipina et al. (2005)	Main effect of age on: - Consecutive correct responses, *F*(1,442) = 38.59, *p* <.01, *B* = 0.57; - Non-perseverative errors, *F*(1,442) = 28.38, *p* <.01, *B* = −0.38. Perseverative errors remained stable across ages, *F*(1,442) = 2.65, *p* <.10, *B* = − 0.04.	Not established
Miller & Marcovitch, 2015	B trials were repeated until children retrieved the object correctly at B location twice or refused to continue to reach	Passing A trials, searching correctly on B1/correct switching	14 M: A trials - *M*(*SE*) = 0.72(0.07), range = 0–1; First B trial - *M*(*SE*) = 0.09(0.05), range = 0–1. 18 M: A trials - *M*(*SE*) = 0.96(0.03), range = 0–1; First B trial - *M*(*SE*) = 0.29(0.07), range = 0–1.	Passing A trials: significant effect of age - McNemar *X*^2^(1, *n* = 47) = 6.67, *p* =.01. First B trial: Not significant - McNemar *X*^2^(1, *n* = 32) = 2.50, *p* =.11.	Children who did not pass the A-trial phase were excluded from the B-trial performance measures, as they did not undergo a reversal trial
Mulder et al., 2020	Incorrect responses on two consecutive A trials in a maximum of four attempts	Correct switching	42% passed the first switch trial, 54% passed the second switch trial, 21% passed the third switch trial, and 13% passed the fourth switch trial.	Not applicable	Not established
Nampijja et al., 2012	End of trials	Total correct	*M*(*SD*) = 4.01(2.36), range = 0–10	Not applicable	All ten trials
Nimmapirat et al., 2024	Four consecutive incorrect trials	Perseverative errors	12M: *M*(*SD*) = 31.22(51.85); range = 0–200 18M: *M*(*SD*) = 74.44(75.83); range = 0–200	No significant correlation between performance at 12 M and 18M (*r* =.04, *p* >.05)	Not established
Noland et al., 2003	Incorrect responses in two out of three reversal trials	Scale	A-not-B rating > 4: 33% A-not-B rating: *M*(*SD*) = 2.6(1.5)	Not examined	Pass the warm-up phase
Nolvi et al., 2018	Incorrect responses in more than a half of the trials	Total correct	*M*(*SD*) = 4.9(3.9)	Not applicable	Codable behavior in at least the first three trials
Phillips et al., 2011	Not established	Total correct	*M*(*SD*) = 0.95(0.08)	Not applicable	Not established
Prado et al., 2016a	End of trials	Total correct	*M*(*SD*) = 6.3(2.3)	Not applicable	Not established
Prado et al., 2016b	End of trials	Total correct, perseverative errors, and completion of all trials	-	Not applicable	Not established
Prado et al., 2016c	End of trials	Total correct, perseverative errors, and completion of all trials	-	Not applicable	Not established
Prado et al., 2016d	End of trials	Total correct, perseverative errors, and completion of all trials	Total correct: *M*(*SD*) = 0.05(0.97) Perseverative errors: *M*(*SD*) = 0.02(1.01)	Not applicable	Not established
Prado et al., 2018	End of trials	Total correct and perseverative errors	-	Not applicable	All ten trials
Pushina et al., 2005	Two incorrect responses in a certain delay	Maximum delay	-	Main effect of age: (*F*(3,106) = 18.24, *p* <.001).	Not established
Pyykko et al., 2020	End of trials	Correct switching	*M*(*SD*) = 1.06(1.10), in possible range of 0–4	Not applicable	All ten trials
Shinya & Ishibashi, 2022	End of trials	Looking time, proportion of correct anticipatory looks pre e pos-switch	-	Not applicable	Inattentiveness
St. John et al., 2016	A maximum of 24 trials and 4 reversal trials administered	Total correct, perseverative errors (proportion)	cf. St. John et al. (2016)	Main effect of age on proportion of total correct: *X*^2^ = 67.73, *p* <.001, and on proportion of perseverative errors: *X*^2^ = 6.79, *p =*.009.	Not established
Sun et al., 2009	Completion of all trials on the 3 cup task or failed 3 consecutive trials	Total correct, number of times the child continued to play with the lid more than 2 s in correct trials (proportion)	Total correct: *M*(*SD*) = 17.33(0.86), possible range of 0–48 Number of times played with lid more than 2 s in correct trials: *M*(*SD*) = 0.37(0.04)	Not applicable	Not established
Tofail et al., 2018	End of trials	Total correct	*M*(*SD*) = 0.00(1.00), possible range of 0–10	Not applicable	Not established
Toth et al., 2008	2 reversals followed by two consecutive correct trials at 12 s or 24 trials	Total correct, perseverative errors, searching correctly on B1	Total correct: 59.9 and 59.5% of the children sucessfully retrieved the object with a 5 s and 12s-delay, respectively; 49.8% of the children successfully completed the ABID; Perseverative errors (Score on reversal trials): 5 s delay (0.54), 12 s delay (0.47), ABID (0.35); Score on B1: 5 s delay (0.33), ABID (0.33).	Correct overall at 12 s and Correct reversal trials at 12 s was associated with age (*r* =.215, *p* <.05, and *r* =.195, *p* <.05, respectively)	Pass the warm-up phase
	3 reversals followed by two consecutive correct trials or 14 trials	Total correct, perseverative errors, searching correctly on B1		No associations between ABID performance and age (*p* >.05).	Not established
Tsetlin et al., 2012	Incorrect responses in a certain delay	Maximum delay at which children performed correctly twice	-	Not applicable	Not established
van de Weijer-Bergsma et al., 2010	Incorrect responses in two out of three attempts in an item	Scale	*7M*: Looking - *M*(*SD*) = 1.74(0.99), % of infants passing A-not-B reversal: 1.4%; Reaching - *M*(*SD*) = 1.53(1.05), % of infants passing A-not-B reversal: 2.8%. *10M*: Looking - *M*(*SD*) = 2.81(1.36), % of infants passing A-not-B reversal: 22.1%; Reaching - *M*(*SD*) = 3.94(2.01), % of infants passing A-not-B reversal: 45.6%. *14M*: Reaching - *M*(*SD*) = 6.61(2.16), % of infants passing A-not-B reversal: 86.4%.	Reaching at 7 M significantly associated with reaching at 10M (*r* =.226, *p* <.05). Looking at 10 M significantly correlated with reaching at 14M (*r* =.296, *p* <.01). Reward retrieval on reversal trials (i.e., item 4 of the scale) increased significantly from 7–10 M and 10–14 M (both *p* <.001).	Pass the warm-up phase
	Incorrect responses in an item twice				Not established
van de Weijer-Bergsma et al., 2016	Incorrect responses in two out of three attempts in an item	Scale	-	Performance at 7 M was significantly correlated with performance at 10M (*r* =.226, *p <*.05) and performance at 10 M was significantly associated with performance at 14M (*r* =.296, *p <*.05).	Pass the warm-up phase
	Incorrect responses in an item twice		-		Not established
Wang et al., 2021	End of trials	Profile of behavior	-	Not applicable	Not established
Wiebe et al., 2010	4 reversals administered	Performance on the first and second post-reversal trials (proportion), number of reversal sets administered, number of trials administered before the criterion of two consecutive correct trials was attained.	*15M*: Proportion correct 1 st post-reversal trial: *M*(*SD*) = 0.24(0.38), range = 0–1; Proportion correct 2nd post-reversal trial: *M*(*SD*) = 0.22(0.33), range = 0–1; Total reversals administered: *M*(*SD*) = 1.57(0.94), range = 0–4; Trials to criterion: *M*(*SD*) = 3.56(2.06), range > 1. *20M*: Proportion correct 1 st post-reversal trial: *M*(*SD*) = 0.30(0.36), range = 0–1; Proportion correct 2nd post-reversal trial: *M*(*SD*) = 0.40(0.36), range = 0–1; Total reversals administered: *M*(*SD*) = 2.23(1.14), range = 0–4; Trials to criterion: *M*(*SD*) = 4.25(2.99), range > 1.	Main effect of age only for completion of more reversal trials, *F*(1, 29) = 6.17, *p* <.02.	Children who never reached criterion were assigned a score of 0 on the post-reversal measures to permit their inclusion in the analyses.
Zmyj et al., 2015	Successfully finding the object in B	Number of A-search trials before the first B response.	*M*(*SD*) = 1.4(1.7), range = 0–5	Not applicable	Not established

Data on performance refers only to control or reference groups, unless it is clearly stated otherwise

^a^ Infants were classified as *partial* when obtaining a partially-hidden toy; *complete* when successfully retrieving a completely hidden toy; A-not-B, when able to retrieve objects from location A but not location B; and *AB* when able to switch to well B after 2 correct trials at A

### Researchers’ assessment interests when administering the A-not-B task

In the present review, we aimed to examine researchers’ usage of the A-not-B task as a tool for assessing EF and related cognitive concepts. The majority of studies primarily focused on assessing global EF (*n* = 26; e.g., Blankenship et al., 2019), followed by the core components of working memory (*n* = 19; e.g., Pushina et al., 2005), inhibitory control (*n* = 13; e.g., Hendry et al., 2022), and cognitive flexibility (*n* = 3; e.g., Borioni et al., 2022). Additionally, a more recent study (Nimmapirat et al., 2024) explored cool EF. Among the studies that examined EF-related concepts, four specifically investigated attention/distractibility (e.g., Sun et al., 2009), while one focused on executive control (Wiebe et al., 2010).

### Most common procedures administered

i) General overview

Several variations of the A-not-B task have been commonly used in research to assess EF and related cognitive processes. These variations differ in the specific procedural modifications made to assess different aspects of the child’s cognitive abilities. Below, the four most frequently used administration procedures are described: the standard A-not-B task, the procedures based on Clearfield and colleagues (2006), the A-not-B task with invisible displacement, and the A-not-B task with multiple hiding locations.

A. Standard A-not-B task

The majority of the studies, particularly those focused on the first year of life (*n* = 21, e.g., Cuevas et al., 2012a), have employed the standard A-not-B task (*n* = 41; e.g., Abubakar et al., 2013). In this procedure, the experimenter repeatedly hides an object in location A - in full view of the infant – while the infant observes the hiding event. Following several successful retrievals from location A, the experimenter switches the location and hides the object in location B. Once more, the experimenter encourages the infant to search for and retrieve the object. Some studies have made adaptations to the procedure in terms of delay, in which authors included an associated delay that either is fixed according to the children’s age (e.g., Hogan et al., 2013) or varies according to the infants’ performance (e.g., Holmboe et al., 2008); or in terms of establishing a fixed order of hidings and patterns of delay levels (e.g., Karonen et al., 2023).

B. Procedures based on Clearfield et al. (2006)

The second most used procedure referred to the procedures based on Clearfield and colleagues (2006) (*n* = 6; e.g., Clearfield & Niman, 2012). Originally, researchers introduced this modified version of the A-not-B, that has a training procedure where there is gradual change in the positioning of the locations (from close to the infant until the hiding event is done on the A location), so that all infants were coaxed in reaching towards the A locations and could build a stronger motor memory, one of the explanatory factors of perseveration (Thelen et al., 2001).

The existing six studies that assessed EF using the procedures of Clearfield et al. (2006) on the A-not-B task have been more used in children up to 12 months (*n* = 4; e.g., Johansson et al., 2014), followed by studies that assessed children between the ages of 13 and 24 months (*n* = 1; Borioni et al., 2022) and both age groups (*n* = 1; Johansson et al., 2015).

In this procedure, two lids are placed on a display box, each corresponding to locations A and B, in front of the infant. Initially, the object is hidden under location A for six consecutive trials. On the first trial, location A is positioned closer to the infant than location B, at the front edge of the box. After a delay, the box is moved forward into the infant’s reaching space, allowing the infant to reach for the hidden object. This process is repeated across multiple trials, with the position of location A gradually being moved further back on the display box until, by trials A4 to A6, location A is aligned with location B. In the critical switch trial, the experimenter shifts the focus to location B by waving it to attract the infant’s attention. After another moment of delay, the box is moved forward again, and the infant is allowed to reach for the object. In the original procedure, six A trials (pre-switch) are followed by one B trial (post-switch). However, some studies have made slight adjustments, such as using six A trials and two B trials (e.g., Johansson et al., 2014) or reducing to four A trials and one B trial (e.g., Wang et al., 2021). Shinya and Ishibashi (2022) used a different version of this procedure, resorting to four A trials and two B trials, in a looking format of the task, where the progressive movement of locations was not introduced.

C. A-not-B task with Invisible Displacement

Another commonly used procedure refers to the A-not-B task with invisible displacement (ABID) (*n* = 5; e.g., Wiebe et al., 2010), which assessed children in the second year of life (e.g., Cuevas & Bell, 2014). This procedure, originally designed by Diamond et al. (1997), is a protocol in which the experimenter first places an object under a container (Location A) in plain sight of the infant, allowing them to see where the object is hidden. Then, a barrier is placed between the hidden object and the infant to prevent the infant from fully maintaining their gaze upon the toy; afterward, the experimenter can either move it to the left or right and change position to the second location.

D.  A-not-B task with multiple hiding locations

The least used administration procedure was the A-not-B with multiple hiding locations (ABMHL; *n* = 4; e.g., Mulder et al., 2020). All studies conducting the ABMHL version of the task were focused on children in the second year of life (e.g., Gago Galvagno et al., 2019). This procedure is another variation of the standard A-not-B task, which expands the number of hiding locations beyond two. The outer locations are selected as the A and B locations, where the experimenter would hide the object, while the center locations are considered neutral.

(ii) Per domain of assessment

Global EF was mainly assessed resorting to the standard version of the A-not-B task (*n* = 18; e.g., Blankenship et al., 2019), followed by the ABMHL (*n* = 4; e.g., Gago Galvagno et al., 2019) and the ABID procedure (*n* = 3; e.g., Cuevas & Bell, 2014). Of these, two studies resorted to both the standard and the ABID version, according to the children’s age (van de Weijer-Bergsma et al., 2010, 2016). Only one study resorted to the procedures proposed by Clearfield et al. (2006) (Johansson et al., 2015).

All but one of the studies assessing working memory have resorted to the A-not-B standard version (*n* = 18; e.g., Pushina et al., 2005), with the remaining employing the ABID version (Toth et al., 2008). Similarly, three examining attention have administered the A-not-B standard version (e.g., Sun et al., 2009), while the other study opted for the procedures based on Clearfield et al. (2006) (Shinya & Ishibashi, 2022).

Inhibitory control was assessed using the standard version of the A-not-B task (*n* = 11; e.g., Hendry et al., 2022), followed by the ABID (*n* = 1; Toth et al., 2008) and procedures by Clearfield et al. (2006) (*n* = 1; Wang et al., 2021). Similar to global EF, Toth et al. (2008) resorted to both the standard and ABID versions of the A-not-B task to assess working memory and inhibitory control.

The three studies tapping cognitive flexibility resorted to Clearfield et al. (2006)’s procedures (e.g., Borioni et al., 2022).

Cool EF (*n* = 1; Nimmapirat et al., 2024) was assessed using the standard version. The only study aiming to capture executive control employed the ABID version (Wiebe et al., 2010).

### A-not-B administration procedures

The administration of the A-not-B task, while generally consistent in its underlying structure, can vary depending on the specific goals of the study and the cognitive components being assessed. Below, the standard procedures for administering the A-not-B task and its variations are described to provide a clearer understanding of how researchers implement these tasks to assess EF and related cognitive processes.

A. A-not-B Task Format

(i) General overview

In terms of task format, reaching is the most chosen A-not-B task format (*n* = 41; e.g., Karonen et al., 2023) for all age groups, followed by the looking version (*n* = 11; e.g., Shinya & Ishibashi, 2022) – which was mostly selected to assess infants up to 12 months of age (e.g., Cuevas et al., 2012b) – and finally, resorting to both formats (*n* = 4; e.g., Frick et al., 2018).

(ii) Per domain of assessment

Across domains of assessment, global EF included 20 studies with the use of the reaching format (e.g., Abubakar et al., 2013), three resorted to the looking format (e.g., Blankenship et al., 2019), and another three employed both formats (e.g., Frick et al., 2018). Similarly, the reaching format was the most chosen for attention (*n* = 3; e.g., Jia et al., 2021; vs. looking: *n* = 1; Shinya & Ishibashi, 2022), working memory (*n* = 13; e.g., Pyykko et al., 2020; vs. looking: *n* = 6; e.g., Cuevas et al., 2012a), inhibitory control (*n* = 11; e.g., Holmboe et al., 2018; vs. looking: *n* = 2; e.g., Bell & Wolfe, 2007), and cognitive flexibility (*n* = 2; e.g., Clearfield & Niman, 2012; vs. both formats: *n* = 1; Johansson et al., 2014). Executive control was examined in one study using the reaching format (*n* = 1; Wiebe et al., 2010), while cool EF was assessed using the looking format (*n* = 1; Nimmapirat et al., 2024).

B. Number of hiding locations

(i) General overview

Considering the number of hiding locations, studies across age groups have mostly resorted to the standard two-location version of the A-not-B task (*n* = 47), whereas a few studies have introduced variability in the locations throughout the testing, to increase task complexity (*n* = 4), particularly research focused on infants up to 12 months of age (e.g., Jia et al., 2021). In these studies, the number of hiding locations was not fixed but instead varied across trials; for instance, a study varied between one and two locations (*n* = 1; Phillips et al., 2011), while others (*n* = 3; e.g., Jia et al., 2021) introduced one to three locations during testing. Additionally, some studies have incorporated multiple but fixed hiding locations (*n* = 5; e.g., Mulder et al., 2020), varying from three to five locations.

(ii) Per domain of assessment

The majority of the studies that assessed global EF (*n* = 21; e.g., Cuevas & Bell, 2014; vs. three locations: *n* = 1; Noland et al., 2003; four locations: *n* = 1; Mulder et al., 2020; five locations: *n* = 3; e.g., Carlson et al., 2004), working memory (*n* = 15; e.g., Bell, 2012; vs. one to two locations: *n* = 1; Phillips et al., 2011; one to three locations: *n* = 3; e.g., Jia et al., 2022), inhibitory control (*n* = 11; e.g., Wang et al., 2021; vs. one to two locations: *n* = 1; Phillips et al., 2011; one to three locations: *n* = 1; Jia et al., 2021) have used the A-not-B task with two locations.

All of the studies considering cognitive flexibility (*n* = 3), executive control (*n* = 1), and cool EF (*n* = 1) have resorted to the two-location version of the task.

Finally, three studies have examined attention varying the locations between one and three (e.g., Jia et al., 2021), with another study using two locations (Shinya & Ishibashi, 2022).

III. Delay Between Hiding and Searching and Criteria for Location Switching

(i) General overview

Overall, most studies employed a fixed delay (*n* = 28; e.g., Mulder et al., 2020), with some specifying that the delay was set based on the child’s age or particular goals (e.g., Hogan et al., 2013). However, some studies did not impose any delay (*n* = 7), while others used delays that varied according to the infant’s performance on the task (*n* = 21).

When considering the delays that varied based on the infant’s performance, there was variability in the criteria used. In eleven studies, the delay was adjusted by adding a few seconds after the child completed a certain number of consecutive correct trials or reducing the delay if the child made a number of errors (e.g., Holmboe et al., 2008). Five studies (e.g., Karonen et al., 2023) used a criterion based on task difficulty levels, adjusting the delay according to the child’s performance. If the child provided a certain number of correct responses, they were then allowed to progress to a more difficult level of the task, with an increased delay. Finally, five studies (e.g., Nimmapirat et al., 2024) only introduced a delay when the child completed a certain number of reversal trials correctly.

With respect to participant’s age, it is apparent that a fixed delay was employed mostly in studies with children in the second year of life (*n* = 21; e.g., Miller & Marcovitch, 2015), while a delay that varied according to the children’s performance was mostly used in infants up to 12 months of age (*n* = 15; e.g., Cuevas & Bell, 2011).

As for the criteria for switching between locations, the majority of researchers used two consecutive correct trials as reference (*n* = 35); other studies employed similar criteria, for instance, used three consecutive correct trials (*n* = 2; e.g., Carlson et al., 2004) or chose either two or three consecutive correct trials depending on the children’s age at testing (*n* = 1; Hendry et al., 2022). Another common approach involved using a pattern or sequence of trials (*n* = 10), for example, the procedure outlined by Clearfield et al. (2006). Two studies opted to switch after a certain number of correct responses in location A (e.g., Zmyj et al., 2015). Finally, six studies did not specify the requirements used for switching between locations.

(ii) Per domain of assessment

Concerning the delay parameter, the majority of the studies on global EF (*n* = 17; e.g., Espy et al., 1999a; vs. no delay: *n* = 1; Blankenship et al. 2019; varies according to the performance: *n* = 6; e.g., van de Weijer-Bergsma et al., 2010; delay levels: *n* = 2; e.g., Karonen et al., 2023) and working memory (*n* = 6; e.g., Tofail et al., 2018; vs. no delay: *n* = 3; e.g., Bacher et al., 2017; varies according to the performance: *n* = 3; e.g., Jia et al., 2022; delay levels: *n* = 3; e.g., Sun et al., 2009; delay after criterion is achieved: *n* = 4; e.g., Cuevas et al., 2012a) have used a *fixed delay*. Studies on cognitive flexibility (*n* = 3) and executive control (*n* = 1) also resorted to this type of delay.

Contrarily, no delay has been used in the majority of the studies examining inhibitory control (*n* = 4; e.g., Ishibashi & Moriguchi, 2017; vs. fixed delay: *n* = 3; e.g., Toth et al., 2008; varies according to the performance: *n* = 3; e.g., Holmboe et al., 2008; delay levels: *n* = 2; e.g., St. John et al., 2016; delay after criterion is achieved: *n* = 1; Cuevas et al., 2012b). The single study on cool EF has employed a delay after a criterion is achieved (Nimmapirat et al., 2024).

For attention, two studies have employed a delay that varied according to the children’s performance (e.g., Jia et al., 2021), one using delay levels (Sun et al., 2009), and the remaining (Shinya & Ishibashi, 2022) used a fixed delay.

Regarding the criteria for switching between locations, two consecutive correct trials were selected for the majority of studies concerning *global EF* (*n* = 19; e.g., Espy et al., 1999b; vs. established sequence: *n* = 4; e.g., Nolvi et al., 2018; three consecutive correct trials: *n* = 2; e.g., Carlson et al., 2004; until the child found the object in B three times: *n* = 1; Gago Galvagno et al., 2019), *working memory* (*n* = 12; e.g., Tsetlin et al., 2012; vs. not established: *n* = 5; e.g., Colombo et al., 2014; established sequence: *n* = 1; Pushina et al., 2005; four successful retrievals from A: *n* = 1; Zmyj et al., 2015), and *inhibitory control* (*n* = 7; e.g., Holmboe et al., 2018; not established: *n* = 4; e.g., Ishibashi & Moriguchi, 2017; established sequence: *n* = 1; Wang et al., 2021; two or three consecutive correct trials: *n* = 1; Hendry et al., 2022). The studies examining cool EF (*n* = 1) and executive control (*n* = 1) have also used two consecutive correct trials.

For attention, this was not the case, as two studies did not define a criterion (e.g., Jia et al., 2022), one established a sequence (Shinya & Ishibashi, 2022), and another used two consecutive correct trials (Sun et al., 2009). Finally, for cognitive flexibility, the three studies have used an established sequence.

D. Pre-Test Training and Test Conclusion Criteria

(i) General overview

As for the training phase, only ten out of 56 administration procedures implemented a pre-test training procedure for the children (e.g., Wiebe et al., 2010). For example, in a study by Toth et al. (2008), the examiner hid the object in one of two locations while the child watched. If the children could find the hidden object successfully, they would proceed to the test phase.

Several criteria were used to determine the conclusion of testing: the completion of trials or sequences (*n* = 22; e.g., Frick et al., 2018), reaching the maximum number of trials or reversals (*n* = 4; e.g., St. John et al., 2016), the occurrence of a specific number of (consecutive) errors or errors during reversal trials (*n* = 18; e.g., Bacher et al., 2017), or the completion of all trials or occurrence of incorrect responses (*n* = 1; Sun et al., 2009). Other criteria included successfully locating the object (*n* = 4; e.g., Miller & Marcovitch, 2015), such as finding the object in location B once or twice. Finally, seven studies did not specify criteria for determining the end of testing.

(ii) Per domain of assessment

Most studies on *global EF* (*n* = 20; e.g., Cuevas & Bell, 2014; vs. existence of pre-test training: *n* = 6; e.g., Miller & Marcovitch, 2015), *working memory* (*n* = 16; e.g., Cuevas & Bell, 2011; vs. existence of pre-test training: *n* = 3; e.g., Pushina et al., 2005), and *inhibitory control* (*n* = 11; e.g., Wang et al., 2021; vs. existence of pre-test training: *n* = 2; Toth et al., 2008) have *not trained* infants before the moment of testing. Similarly, attention (*n* = 4), cognitive flexibility (*n* = 3), and cool EF (*n* = 1) have not included training in their protocol. On the other hand, the single study on executive control trained its sample of children (Wiebe et al., 2010).

End of testing would occur when, for *global EF* (*n* = 11; e.g., Abubakar et al., 2013; vs. a certain amount of incorrect responses: *n* = 9; e.g., Lipina et al., 2005; finding object in reversal trials: *n* = 3; e.g., Carlson et al., 2004; not established: *n* = 3; e.g., Hogan et al., 2013), *inhibitory control* (*n* = 5; e.g., Hendry et al., 2022; administration of reversal and/or no. trials: *n* = 3; e.g., St. John et al., 2016; a certain amount of incorrect responses: *n* = 2; e.g., Cuevas et al., 2012b; not established: *n* = 3; e.g., Colombo et al., 2014), and *cognitive flexibility* (*n* = 3), a *fixed number of trials were administered*.

After a *certain number of incorrect responses* occurred, testing would end for *working memory* (*n* = 8; e.g., Bacher et al., 2017; administration of reversal and/or no. trials: *n* = 3; Toth et al., 2008; end of trials: *n* = 2; e.g., Pyykko et al., 2020; finding object in reversal trials: *n* = 1; Zmyj et al., 2015; completion of trials or incorrect responses: *n* = 1; Sun et al., 2009; not established: *n* = 4; e.g., Jia et al., 2021) and *cool EF* (*n* = 1).

For attention, two studies have not defined a criterion for testing termination (e.g., Jia et al., 2022), one established a number of trials (Shinya & Ishibashi, 2022), and another used the completion of all determined trials or a certain number of incorrect responses (Sun et al., 2009). Finally, for executive control, Wiebe et al. (2010) have resorted to the number of reversals that were administered.

### Researchers’ scoring methods of the children’s performance

Scoring infants’ performance on the A-not-B task has varied across studies, with researchers employing different methods to attribute an EF score. The details of researchers’ scoring methods will be presented per domain of assessment and per category of assessment (please see Table 3 for a description of the scoring methods, available in the supplementary materials). In the third subsection, results concerning the role of age are presented.

A. Per domain of assessment

When focusing on global EF, the majority of researchers have resorted to total correct (*n* = 12; e.g., Cuevas & Bell, 2014), followed by a number of perseverative errors (*n* = 11; e.g., Abubakar et al., 2013), and to a scale (*n* = 5; e.g., Noland et al., 2003). Cool EF was assessed only through the number of perseverative errors (Nimmapirat et al., 2024).

For working memory, total correct continued to be the scoring method with the most expression (*n* = 12; e.g., Cuevas & Bell, 2011), followed by perseverative errors (*n* = 5; e.g., Bell, 2012) and non-perseverative errors (*n* = 3; e.g., Cuevas et al., 2012a), and correct switch/finding object in B (*n* = 3; e.g., Pyykko et al., 2020).

When interested in inhibitory control, total correct was the most used measure (*n* = 5; e.g., Ishibashi & Moriguchi, 2017), followed by perseverative errors (*n* = 4; e.g., Toth et al., 2008), correct switch/finding object in B1 (*n* = 3; e.g., Hendry et al., 2022), and maximum (observed) delay (*n* = 3; e.g., Holmboe et al., 2008). It is important to add that the scoring method “maximum observed delay” differs from “maximum delay” in the sense that the latter only considers the delay that was set to that given trial; with maximum observed delay referring to the delay between the moment the object is hidden and when the infants effectively search for the object.

Three studies assessed cognitive flexibility, and used profiles of behaviour where infants were categorised into different groups based on their performance or search responses (*n* = 2; e.g., Johansson et al., 2014), followed by total correct, perseverative errors, percentage of infants reaching A on B1, and speed (Borioni et al., 2022; Clearfield & Niman, 2012). For instance, Johansson et al. (2014) categorised children into four groups based on their looking and reaching responses: (i) correct looking/reaching response, (ii) incorrect looking/reaching response, (iii) no search, and (iv) searching at both locations. Similarly, Clearfield and Niman (2012) grouped children into (1) reaching correctly, (2) reaching randomly, or (3) perseveration.

Finally, for EF-related concepts, attention/distractibility was assessed mainly through the number of times the infant continued to play with the cup (*n* = 3; e.g., Jia et al., 2022), i.e., when an infant reached for the hidden location of the toy correctly but preferred to play with the lid for more than 2 s; and executive control through the performance on the first and second post-reversal trials, number of reversal sets administered, and number of trials administered before the criterion of two consecutive correct trials was obtained (Wiebe, 2010).

B. Per categories of assessment

This subsection outlines the scoring methods used for assessing EF and related concepts, categorized according to different assessment approaches, such as (a) accuracy, for studies that used correct/incorrect procedures; (b) delay, when the performance in the task was scored from the delay they could endure; (c) distractibility, for studies that were interested in understanding whether the child became distracted during the task; (d) looking behavior, when measures as looking time were used; and (e) others, when the procedures employed were diverse and could not find a fit in the previous categories. The most common assessment category focused on infants’ accuracy on the A-not-B task (*n* = 68). In this category, the most used measures concern the total correct trials (*n* = 26), perseverative errors (*n* = 21), non-perseverative errors (*n* = 5), and correct switches (*n* = 4). Noteworthy to mention is the fact that Holmboe et al. (2008) have opted to use a cumulative measure to compare participants with different trial completion, which refers to the sum of observed delays for correct change trials divided by total correct trials.

Additionally, in the delay category, researchers employed measures such as maximum delay (*n* = 3; e.g., Colombo et al., 2014), maximum observed delay (*n* = 1; Holmboe et al., 2008), and maximum delay at which the infant performed correctly twice (*n* = 1; Tsetlin et al., 2012).

The distractibility category specifically concerns the number of times the infant interacted with the lid for more than 2 s (*n* = 3; e.g., Jia et al., 2021). The looking behavior category included measures such as the proportion of looking time (*n* = 2; e.g., Johansson et al., 2015) and the proportion of correct anticipatory looks (*n* = 1; Shinya & Ishibashi, 2022). Finally, another category was created to include the following scoring methods. These included scale (*n* = 5; e.g., van de Weijer-Bergsma et al., 2010), whether children completed all the trials (*n* = 3; e.g., Prado et al., 2016b), a profile of behaviours (*n* = 3; e.g., Hogan et al., 2013), the proportion of infants performing different types of performance (*n* = 1; Johansson et al., 2014), and speed (*n* = 1; Borioni et al., 2022). One notable scale for assessing global EF was developed by Bell and colleagues (Bell & Adams, 1999; Bell & Fox, 1992) and later used in a study by Noland et al. (2003). This scale ranged from 0 (performance never merited a single reversal trial) to 7 (failed A-not-B criteria at an 8-sec delay) and was coded depending on infants’ performance at different delay levels. In addition, as an example of a behavioural profile approach, Hogan et al. (2013) categorized infants into four groups - partial, complete, A-not-B or AB – based on their ability to locate a hidden toy. Hogan et al. (2013)’s four classifications depended on whether the infant could retrieve the object when it was partially or fully hidden and whether they could successfully find it in both locations A and B.

C. The role of age on children’s performance

Across the 56 included administration procedures, 22 investigated the role of children’s age in task performance. A significant effect of age was reported in 19 studies, whereas no significant effect was found in three studies. The reports that did not find an age effect measured children’s performance using perseverative errors (*n* = 2; e.g., Lipina et al., 2005), profile – control group (*n* = 1; Hogan et al., 2013), total correct (*n* = 2; e.g., Ishibashi & Moriguchi, 2017), accuracy in the first B trial (*n* = 2; e.g., Miller & Marcovitch, 2015), accuracy in the second B trial (*n* = 1; Wiebe et al., 2010), and number of trials necessary to reach criterion (*n* = 1; Wiebe et al., 2010), although some these methods were also in studies reporting significant effects.

On the other hand, age emerged as being significantly correlated with, and predictive of, total correct (*n* = 6; e.g., Toth et al., 2008), performance measured by a scale (*n* = 4; e.g., van de Weijer-Bergsma et al., 2016), perseverative errors (*n* = 3; e.g., St. John et al., 2016), non-perseverative errors (*n* = 2; e.g., Lipina et al., 2005), maximum delay (*n* = 2; e.g., Pushina et al., 2005), number of consecutive correct responses (*n* = 2; e.g., Lipina et al., 2005), maximum error run (*n* = 1; Abubakar et al., 2013), perseverative runs (*n* = 1; Espy et al., 1999b), profile (*n* = 1; Hogan et al., 2013), looking time (*n* = 1; Johansson et al., 2015), passing A trials (*n* = 1; Miller & Marcovitch, 2015), and number of reversals administered (*n* = 1; Wiebe et al., 2010). Clearfield and Niman (2012) also found the effect of age, when interacting with socioeconomic status, for perseveration. Finally, in one study, the scores of different tasks tapping EF were combined, with age emerging as being correlated with children’s performance (Gago Galvagno et al., 2019).

When considering the domain of assessment, age was a significant predictor of global EF (*n* = 11; e.g., van de Weijer-Bergsma et al., 2016), working memory (*n* = 6; e.g., Pushina et al., 2005), inhibitory control (*n* = 3; e.g., Colombo et al., 2014), cognitive flexibility (*n* = 1; Clearfield & Niman, 2012), and executive control (*n* = 1; Wiebe et al., 2010). Age was not found for two studies assessing inhibitory control (e.g., Ishibashi & Moriguchi, 2017), one examining working memory (Toth et al., 2008), and for the single study of cool EF (Nimmapirat et al., 2024).

### Participation inclusion criteria

(i) General overview

Most studies did not provide information regarding participation inclusion criteria (*n* = 34). Among the remaining studies, the inclusion criteria varied considerably. Completion of all trials was the most frequent (*n* = 7; e.g., Espy et al., 1999a), with other studies having used similar criteria, which referred to including participants in the analysis that completed at least one trial (*n* = 1; Frick et al., 2018), have codable behavior on the first three trials (*n* = 1; Nolvi et al., 2018), more than a half of the trials (*n* = 1; Carlson et al., 2004), or a minimum of 10 trials (*n* = 2). The studies that included participants with a minimum of 10 trials also required the administration of change trials (*n* = 1; Holmboe et al., 2018), and one correct reach for each location (*n* = 1; Hendry et al., 2022). Out of the ten studies with a pre-test phase, two clearly stated that passing the “warm-up” phase was necessary to be included in the study (e.g., Noland et al., 2003), and another two could be presumed since the A-not-B testing only began after the child could retrieve the object in the training phase (e.g., van de Weijer-Bergsma, 2010). The existence of correct and incorrect trials was also required in one study, combined with a minimum of three minutes of assessment (*n* = 1; Bacher et al., 2017). Similarly, the study of Johansson et al. (2015) used the baseline criteria of infants performing two times correctly in location A and one in location B, and having longer looking times to the correct location. Researchers also expressed concern about infants exhibiting a strong side bias (*n* = 1; Johansson et al., 2014), which was evident in infants who consistently reached for and looked toward the incorrect hiding location during all A trials and continued searching on the same side during the B trials; and being inattentive (*n* = 1; Shinya & Ishibashi, 2022) with the looking time being above or below 3 standard deviations. Miller and Marcovitch (2015) only considered children for the B-trial performance measures if they were able to pass the A-trial phase. Finally, a score of 0 was attributed to infants who did not reach the required criteria, so they could be included in the analysis (*n* = 1; Wiebe et al., 2010).

(ii) Per domain of assessment

As mentioned, the majority of the studies have not established a criterion for the inclusion of participants in the analysis. This is the case of *global EF* (*n* = 13; e.g., Prado et al., 2016b; vs. completion of all trials: *n* = 5; e.g., Espy et al., 1999b; completion of a certain no. of trials: *n* = 4; e.g., Frick et al., 2018; pass the warm-up phase: *n* = 3; Noland et al., 2003; passing A-trial phase to be included in the B-trial performance measures: *n* = 1; Miller & Marcovitch, 2015), *working memory* (*n* = 16; e.g., Bell, 2012; vs. completion of all trials: *n* = 1; Tofail et al., 2018; existence of correct and incorrect trials and trials lasting longer than 3 min: *n* = 1; Bacher et al., 2017; pass the warm-up phase: *n* = 1; Toth et al., 2008), *inhibitory control* (*n* = 9; e.g., Bell & Wolfe, 2007; vs. completion of a certain number of trials: *n* = 2; e.g., Holmboe et al., 2018; completion of all trials: *n* = 1; Ishibashi & Moriguchi, 2017; pass the warm-up phase: *n* = 1; Toth et al., 2008), *cognitive flexibility* (*n* = 2; vs. infants not showing a strong side bias: *n* = 1; Johansson et al., 2014), and *attention* (*n* = 3; e.g., Sun et al., 2009; vs. inattentiveness: *n* = 1; Shinya & Ishibashi, 2022). For cool EF, no criterion was defined (*n* = 1). Finally, when assessing executive control, Wiebe et al. (2010) established that children who did not meet a certain criterion were assigned a score of 0 on the post-reversal measures to permit their inclusion in the analyses.

## Discussion

The A-not-B task, originally developed to assess object permanence in children (Piaget, 1954), has more recently been adapted to provide valuable insights into early EF development, which has critical implications for children’s later development (e.g., Holmes et al., 2016; Zelazo & Carlson, 2020). However, the use of various administrational procedures of the A-not-B task to assess EF in children up to 24 months of age presents several challenges in terms of result interpretation. Variability in task design and procedural differences across studies can significantly influence outcomes, making it difficult to draw consistent conclusions.

To address these issues, in this review, we examined the administration procedures of the A-not-B task as a measure of EF and related cognitive concepts in this age group. Fifty-three reports, which were published from 1999 to 2024, were identified as being eligible. Notably, all but two were published between 2003 and 2024, which clearly shows that resorting to the A-not-B task to assess EF in the first two years of life is a recent phenomenon, with this task still proving to be a fit and valuable resource despite its original purpose.

Our findings highlight considerable methodological diversity across studies. Variations were observed in task format (reaching vs. looking), number and configuration of hiding locations, delay intervals, criteria for switching locations, pre-test training, test termination rules, and scoring methods—including total correct responses, perseverative errors, behavioural profiles, and attention or distractibility measures. Participation inclusion criteria were also inconsistent. Such heterogeneity can significantly affect the cognitive and regulatory demands placed on infants, potentially impacting performance and the interpretation of EF abilities. These findings underscore the need for greater standardization and transparency in A-not-B administration and scoring to improve comparability across studies and strengthen the utility of the task for research on early EF.

Amidst procedural heterogeneity, some commonalities emerged. Most studies assessed global EF or its core components, primarily working memory and inhibitory control, consistent with the developmental trajectory of EF, as cognitive flexibility develops more prominently during preschool years and builds on working memory and inhibitory control (Garon et al., 2008; Cuevas & Bell, 2014). EF-related constructs such as executive control and executive attention were less frequently examined. Notably, cool EF was measured at 12- and 18-month time points (Nimmapirat et al., 2024), indicating that this emerging ability can be assessed in infancy and highlighting the potential for further investigation into the early differentiation of EF components.

Regarding specific administration procedures, overall, most researchers resorted to the reaching format of the task (e.g., Clearfield & Niman, 2012) and used the standard two locations (e.g., Zmyj et al., 2015). Most of the procedures did not include a training phase before testing (e.g., Bell, 2012) and used a fixed delay (e.g., Tofail et al., 2018) or varied according to children’s performance (e.g., Tsetlin et al., 2012). The criteria for switching between locations were mostly two consecutive correct responses or the existence of a pattern/sequence of trials (e.g., St. John et al., 2016; Wang et al., 2021); and the ending of testing was determined by an established number of trials or sequences (e.g., Johansson et al., 2014), or if the children committed a certain number of errors (e.g., van de Weijer-Bergsma et al., 2010). Specifically, some researchers terminated the testing when infants made consecutive errors on reversal trials (e.g., Bacher et al., 2017).

To address researchers’ scoring methods, measures were presented by domain and category of assessment. Across domains, total correct responses and perseverative errors were commonly used for global EF, working memory, inhibitory control, and cognitive flexibility. Working memory was also measured via non-perseverative errors, while inhibitory control primarily relied on total correct and perseverative errors, consistent with Bell and Adams (1999), who suggest that working memory demands remain similar across non-reversal and reversal trials, whereas inhibitory demands increase during reversal trials. Attention/distractibility was assessed by the number of times infants continued to play with the cup, and executive control through performance on post-reversal trials, the number of reversal sets, and trials required to reach two consecutive correct responses.

To score children’s performance, researchers have primarily relied on accuracy measures, such as accuracy on reversal and non-reversal trials, and total correct trials (e.g., Cuevas et al., 2012a; Lipina et al., 2005). The delay was also considered with some researchers coding the maximum delay at which the infant performed correctly (e.g., Colombo et al., 2014; Holmboe et al., 2018). Most researchers did not report any data on the criteria for including participants in the analysis. Seven studies required participants to complete all trials (e.g., Ishibashi & Moriguchi, 2017; Pyykko et al., 2020); the remaining presented a wide range of criteria. As mentioned, this lack of standardization in participant inclusion criteria can introduce variability across studies, making it difficult to draw consistent conclusions. To ensure that research findings can be comparable, future studies should clearly state the inclusion criteria used.

The role of age could also impact the administration procedures. A great part of the analyzed studies was carried out with infants up to 12 months (to be expect since the A-not-B error was associated with a small developmental window in the first year). This was followed by research with children between 13 and 24 months of age (some of the studies included older children, but task administration did not vary), and finally, studies that included both age groups. It is noteworthy to mention that the youngest child to be tested with the A-not-B task to assess EF or similar concepts was 5 months old (e.g., Wang et al., 2021).

The looking task format was more commonly used with infants up to 12 months than when compared to older children. This resulted from studies that tested if removing the reaching component (less mature in the first year) would result in less perseveration (Berger, 2004).

Another result worth mentioning refers to the use of multiple locations with older infants. Meta-analyses have shown that the number of locations is a negative predictor of perseverative searching (Marcovitch & Zelazo, 1999; Wellman et al., 1986). This could be explained by the fact that multiple locations, competing for attention, interact with the memory of all the previous reaches to the A location (Smith et al., 1999).

Finally, concerning the delay parameter, although when considering the total sample of studies, the type of delay most frequently selected was the fixed delay, the delay that varied according to the infant’s performance was chosen by the majority of researchers who studied infants in the first year of life (e.g., Colombo et al., 2014). Perhaps this option allowed the task to be more flexible, adaptable, and capture slighter changes in results in a population of smaller children.

Although there was considerable variability in the administration procedures of the A-not-B task, 17 of the 19 studies that tested the role of age reported a significant effect of age. This consistency of the effect of age across heterogeneous methodologies supports the developmental sensitivity of the A-not-B task, indicating that age-related changes are still detectable even when the administration and scoring procedures vary significantly.

### Limitations and directions for future research

Limitations of this work should be considered. Information on some of these studies could not be extracted (e.g., characteristics of the population) since they were not reported in detail, constraining a more thorough analysis. Additionally, studies that did not clearly state that they were employing the A-not-B task were not included in this review, even though they shared similar procedures, which could impair some of the drawn conclusions. Finally, the review includes a small sample of studies, so results should be interpreted cautiously.

Despite these limitations, this review provides an overview of how the A-not-B task has been used to assess EF in children up to 24 months. The observed methodological variability highlights the need for future research to systematically examine how procedural differences—such as task format, hiding locations, delays, switching criteria, and pre-test training—affect performance. Empirical studies identifying the most valid and reliable procedures, combined with collaborative efforts to develop consensus guidelines, could support standardized administration, improve comparability across studies, and strengthen assessment of early EF, ultimately informing developmental research and interventions.

## Supplementary Information

Below is the link to the electronic supplementary material.


Supplementary Material 1 (DOCX 18.4 KB)


## Data Availability

No datasets were generated or analysed during the current study.
