# Supplementary material for: Executive functioning in the first 24 months: A scoping review of the A-not-B task
Source: Psychol Res. 2026 Jul 15;90(4):138. doi: 10.1007/s00426-026-02330-5 (PMC13372870; doi:10.1007/s00426-026-02330-5)
Supplement: Supplementary file 1 — Supplementary Material 1 (DOCX 18.4 KB) [file 426_2026_2330_MOESM1_ESM.docx]

**Supplementary Material**

**Table 3.**

*Description of the scoring measures used across studies*

| **Scoring measure** | **Details** |
| --- | --- |
| Accuracy | Proportion of A trials responded to correctly minus the proportion of B trials responded to correctly |
| Completion of all trials | Whether the child completed the full set of administered trials |
| Correct switching | Total number of correct switches (“A”-to-“B,” or “B”-to-“A”) |
| Cumulative measure in change trials | The sum of the observed delays for all successful change trials in the task for each infant and then dividing the result by the total number of trials completed by that infant |
| Looking time | Looking time at the display during the entire trial and looking at the areas of interest of the two locations |
| Maximum delay | The length of the delay that infants could tolerate before searching incorrectly |
| Maximum delay at which children performed correctly twice | The length of the delay that infants tolerated when they achieved the criterion of two correct responses |
| Maximum delay in change trials | The length of the delay that infants could tolerate before searching incorrectly in reversal trials |
| Maximum error run | Longest string of errors |
| Maximum observed delay | Measured from when the locations were completely covered to when the infant touched one of the locations |
| Mean number of correct searches on all trials | The sum of reaching and looking (across the trials) divided by two |
| Non-perseverative errors | Number of errors committed before the first switch of location |
| Number of A trials to get two consecutive correct responses | Number of A trials required to meet the criterion of two consecutive correct responses |
| Number of A-search trials before first B response | Number of trials in which infants searched at location A before they finally searched at location B |
| Number of correct consecutive responses | Number of consecutive correct trials completed by the child |
| Number of reversal sets administered | Total number of location reversals completed during the task |
| Number of times the child continued to play with the lid more than 2 seconds in correct trials (proportion) | Proportion of trials in which the infant reached the correct location, but played with the cover for more than 2s instead of retrieving the toy. |
| Number of times the child had two consecutive correct responses | Number of times the child achieved two correct responses in succession |
| Number of trials administered before the criterion of two consecutive correct trials was attained | Total trials administered prior to achieving the criterion of two successive correct responses |
| Passing A trials | Retrieving the object at location A correctly three times |
| Percentage of infants reaching A on B1 | Percentage of infants who incorrectly searched at location A during the first trial after the hiding location was switched to B (B1 trial) |
| Performance on the first and second post-reversal trials | Performance on the first two trials after the hiding location is switched |
| Perseverative errors | Number of errors committed after the first switch of location |
| Perseverative runs | Maximum number of trials in a given set of consecutive perseverative errors |
| Profile of behaviours | Classification of participants into groups based on their patterns of responses. |
| Proportion of correct anticipatory looks | Calculated for each trial by dividing the difference between the looking time for the areas of interest of the incorrect and correct boxes by the total looking time for both the areas of interest of the boxes |
| Scale | Use of a scale to categorize children's performance. For instance, Noland et al. (2003) used a scale ranging from 0 - "performance never merited a single reversal trial" to 7 - "failed A-not-B criteria at an 8-sec delay". |
| Searching correctly in B twice | Children were required to search for the object in B until finding it in two occasion. Task completion was defined at the point at which this criterion was reached. |
| Speed | Time spent by the child to retrieve the object |
| Total correct/Proportion correct | Total correct: Sum of correct responses Proportion correct: Number of trials with a correct response divided by the total number of trials presented |
